# The Effectiveness of Hearing Protection Devices: A Systematic Review and Meta-Analysis

**DOI:** 10.3390/ijerph182111693

**Published:** 2021-11-07

**Authors:** Chanbeom Kwak, Woojae Han

**Affiliations:** 1 Laboratory of Hearing and Technology, Research Institute of Audiology and Speech Pathology, College of Natural Sciences, Hallym University, Chuncheon 24252, Korea; cksqja654@gmail.com; 2 Division of Speech Pathology and Audiology, College of Natural Sciences, Hallym University, Chuncheon 24252, Korea

**Keywords:** noise-induced hearing loss, hearing protection, sound attenuation, sound localization, communication

## Abstract

To prevent intensive noise exposure in advance and be safely controlled during such exposure, hearing protection devices (HPDs) have been widely used by workers. The present study evaluates the effectiveness of these HPDs, partitioned into three different outcomes, such as sound attenuation, sound localization, and speech perception. Seven electronic journal databases were used to search for published articles from 2000 to 2021. Based on inclusion criteria, 20 articles were chosen and then analyzed. For a systematic review and meta-analysis, standardized mean differences (SMDs) and effect size were calculated using a random-effect model. The funnel plot and Egger’s regression analysis were conducted to assess the risk of bias. From the overall results of the included 20 articles, we found that the HPD function performed significantly well for their users (SMDs: 0.457, 95% confidence interval (CI): 0.034–0.881, *p* < 0.05). Specifically, a subgroup analysis showed a meaningful difference in sound attenuation (SMDs: 1.080, 95% CI: 0.167–1.993, *p* < 0.05) when to wear and not to wear HPDs, but indicated no significance between the groups for sound localization (SMDs: 0.177, 95% CI: 0.540–0.894, *p* = 0.628) and speech perception (SMDs: 0.366, 95% CI: −0.100–1.086, *p* = 0.103). The HPDs work well for their originally designated purposes without interfering to find the location of the sound sources and for talking between the workers. Taking into account various factors, such as the characteristics of the users, selection of appropriate types, and fitting methods for wearing in different circumstances, seems to be necessary for a reliable systematic analysis in terms of offering the most useful information to the workers.

## 1. Introduction

It is clearly acknowledged that industrial workers and individuals in military service have inevitably faced more noise exposure and that exposure has increased the incidence of noise-induced hearing loss (NIHL) [1,2]. Not only temporary noise exposure of high intensity (i.e., more than 85 dBA) in terms of level of intensity, but also continuous exposure to moderate-intensity noise in terms of frequency can contribute seriously to negative effects on the human auditory system [1,2]. Thus, noise is regarded as a decisive factor producing the hearing loss of workers [3] and consequently accompanying other otologic problems and diseases such as tinnitus, poor hearing ability for situational awareness and communication, and psychological problems such as a lower quality of life [4].

To prevent such exposure to intensive noise in advance and be safely controlled during exposure, hearing protection devices (HPDs) have been under development for several decades. Although HPDs seem to be a passive method of noise control, they are the most practical and good enough when appropriately fitted for a correct size and have received adequate maintenance [5,6]. Unlike their original purpose, however, many workers are frequently reluctant or even do not wear these devices due to discomfort and a tight fit feeling even in a noisy environment [4]. Further, the sound attenuation obtained by wearing the HPDs, which is a key function to prevent NIHL [7], may decrease the ability to identify sound localization [8,9,10] and achieve speech perception [3,11,12,13]. Therefore, many researchers have studied the functions and effects of HPDs whenever a new device is released. Regardless, these studies have produced a problem, namely, how to organize and summarize their different findings, consequently resulting in the suggestion of needing a systematic analysis to achieve the highest level of evidence. Hence, we aimed to evaluate the major functions of the HPDs using systematic review and meta-analysis techniques. More specifically, the effectiveness of HPDs was partitioned to three different functions and effects, including sound attenuation, sound localization, and speech perception, which can give the users clearer expectations about the efficiency of these HPDs when wearing them.

## 2. Materials and Methods

### 2.1. Strategy for Systematic Search

The Preferred Reporting Items for Systematic Reviews and Meta-analysis (PRISMA) 2020 statement [14] and the International Prospective Register of Systematic Reviews (PROSPERO) of Cochrane Collaboration [15] were used as a methodology that commonly processes the systematic search for and meta-analysis of published articles and their review was reported. The PROSPERO registration number was CRD42021276424.

The process applied for the inclusion criteria of articles for the systematic review and meta-analysis [14,15] combined a strategy using participants, intervention, control, outcome measures, and study design (PICOS). Table 1 displays the PICOS criteria used in the present study. On the other hand, our exclusion criteria were modeling studies, engineering design, no research article (e.g., narrative review paper, conference abstract, letters, book and book chapters, magazines, and proceeding paper), and not being written in English.

### 2.2. Article Selection

Seven electronic databases (i.e., Embase, Medline, Pubmed, Web of Science, Science Direct, Scopus, Cumulative Index to Nursing and Allied Health) were used and searched from January 2000 to June 2021 using the key terms, “hearing protection devices” OR “hearing protector” AND “hearing protection devices effects” OR “effects” OR “attenuation” OR “intervention” OR “sound localization” OR “benefits” OR “speech intelligibility”. The terms were always combined to limit identifying duplicate papers.

**Figure 1:**
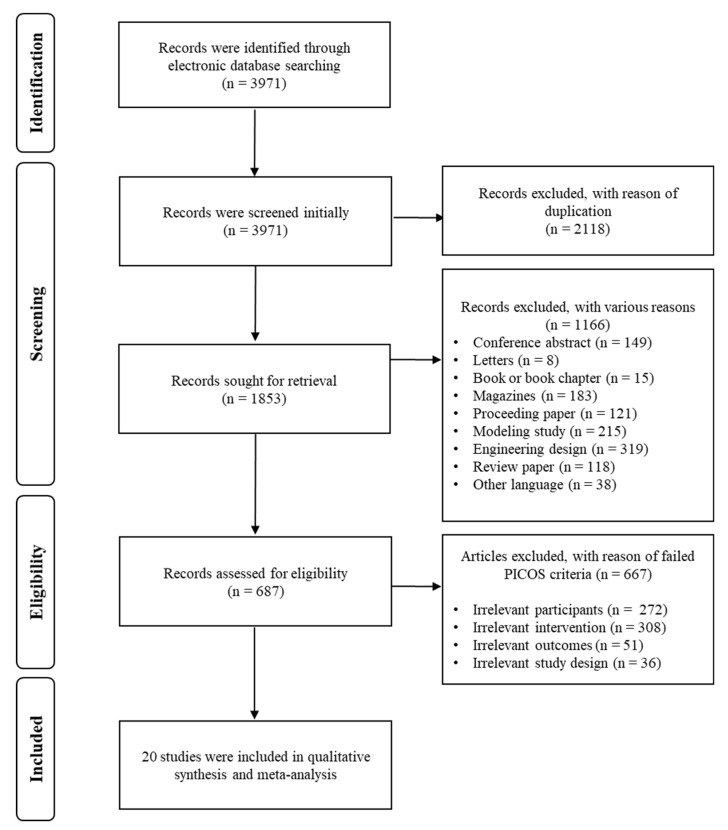
A Preferred Reporting for Items for a Systematic Review and Meta-analysis (PRISMA) flow diagram to explain the inclusion and exclusion process of the current study.

Initially, the number of articles searched in the electronic databases equaled 3971. After eliminating 2118 articles which were met the exclusion criteria due to duplication, 1853 articles remained. As a part of the screening process, their titles and abstracts were confirmed to exclude 1166 records. Then, only 687 articles were accessed to review their full texts at the eligibility stage. Further, because 667 studies failed to meet the PICOS criteria for several reasons (i.e., participants in industrial work and irrelevant outcome measures), a total of 20 articles were included for the specific review. Figure 1 explains each of these steps visually.

### 2.3. Study Quality and Potential Sources of Study Bias

To evaluate both study quality and potential sources of any study bias, the CAMARADES checklist was used [16]. It contained six independent items, namely, randomization (pseudo-randomization of participants allocation, test condition, or materials), controls, sample size calculation (calculation of the appropriate number of study sample produced using power analysis), articles published with peer review, outcome measure, and statement of potential conflict of interests (see Table 2 for details). The scores were calculated using 1, which was assigned by the item (i.e., YES), or 0 (i.e., NO). After providing scores for study quality, each study was summed up at the level of the evidence based on the Grading of Recommendations, Assessment, Development, and Evaluation (GRADE) Working Group Guideline [17]. The study quality scores of selected articles were analyzed by the chi-square test. Simply put, as the level of evidence increased, the results of the study were considered to be valid.

The data contained in the articles were extracted and synthesized for the following content: (1) participants (number, age, sex, and hearing threshold); (2) types of hearing protection devices; (3) study design; (4) the main factor of the experiments (i.e., level of attenuation, ability of sound localization, and performance of speech perception); (5) outcome measures; and (6) major findings. Two authors (C.K. and W.H.) conducted the process independently and then any discrepancies (less than 5%) were resolved by consensus between the two authors.

### 2.4. Meta-Analysis

The included articles were identified to determine whether their data were suitable for meta-analysis. Comprehensive Meta-Analysis software (Ver. 3, Biostat Inc., Englewood, NJ, USA) was used to conduct the meta-analysis. The data collected from the included articles were continuous, and the same outcome measures, mean differences (MDs), were calculated with 95% confidence intervals (CIs). However, when the data having different outcome measures were collected, the effect sizes were calculated using standardized mean differences (SMDs). After the effect sizes were calculated, a summary estimate was examined. As the participants in the reviewed articles were adults, both with and without hearing loss, the random-effect model was used to calculate the effect size and summary estimate. Heterogeneity (genuine differences underlying the results of the studies) [28] across the articles was identified using the Higgins *I^2^*-statistics and Cochran’s Q-test. To quantify heterogeneity, Higgins *I^2^* provided a value from 0 to 100%: 0–25% for low, 25–75% for moderate, and 75–100% for high [28]. Cochran’s Q-test indicated a 95% statistical significance level (*p* < 0.05). The subgroup analysis, which categorized the articles as having three remarkable features (e.g., sound attenuation, sound localization, and speech perception) and meta-regression were considered due to the possibility of high heterogeneity and/or different outcome measures for the reviewed studies.

Reporting bias occurred when the reporting and spreading of the results of studies were influenced by their characteristics and directions of their main findings. The most representative was a publication bias where the studies having statistically significant differences had a larger possibility of publication compared to those studies with no significant differences. Since the publication bias usually produced distorted results for the meta-analysis [29], the funnel plot and Egger’s regression test were used to identify it. The funnel plot is a kind of scatter plot consisting of a y-axis for the sample size of studies and an x-axis for the effect size.

## 3. Results

### 3.1. Study Quality Scores

The scores for study quality, based on the CAMARADES checklists, were analyzed using a chi-square test and R statistical computing software [30]. The mean value of the study quality scores was 3.45 (SD: 0.83, range: 2~5). Then, to identify the goodness of fit for the study quality scores, a chi-square test was also conducted using the R software [30]. There were no significant differences between the study quality scores (χ^2^ = 3.7536, df = 19, *p* > 0.05).

### 3.2. Charactersits of Studies

#### 3.2.1. Participants

The summarized results of the reviewed studies according to the PICOS criteria are displayed in Table 3. The participants included individuals with both normal hearing [4,7,8,9,10,11,12,13,18,19,20,21,22,23,24,25] and hearing loss [3,5,26]. Specifically, Smalt et al. reported on one single individual with a threshold of 30 dB HL in one ear [3] and Abel et al. had 24 older adults with hearing loss [5]. In the study of Giguère and colleagues, the subjects were divided into four groups, e.g., normal hearing, slight-to-mild hearing loss, mild-to-moderate hearing loss, and moderate-to-severe hearing loss [26].

#### 3.2.2. Intervention

Types of HPDs, their functions and effects were evaluated as the intervention. Six of twenty studies conducted a comparison between types of HPDs. For example, in the study by Simpson et al., types of HPDs for earplugs, earmuffs, and a combination of earplugs and earmuffs [24] were compared. On the other hand, fourteen of twenty studies compared two conditions for when to wear and not to wear HPDs by using various scenarios of use, such as earplugs only [3,7,10,12,13,19,20,22], earmuffs only [5,8,9,11], earplugs and/or earmuffs [4,18,21,23,25,26], earplugs with earmuffs [24], and headsets [27]. In terms of functions and effects, only six of twenty studies used passive noise reduction HPDs such as earplugs and earmuffs [5,7,13,20,23,24]. More than half of the studies (fourteen of twenty studies) used HPDs with a function for active noise reduction [3,4,8,9,10,11,12,18,19,21,22,25,26,27].

#### 3.2.3. Controls and Study Designs

It was desirable to ensure that there was high-level evidence (i.e., randomized controlled trials) for the research articles [31]. Additionally, a between-group comparison (i.e., experimental group versus control group) was regarded as an alternative choice to use to confirm the effects of the experiment. In the current review, only three of twenty studies used between-group comparisons [5,8,26], and the other seventeen studies were conducted using repeated measures under various conditions [3,4,7,9,10,11,12,13,18,19,20,22,23,24,25]. In the other view, although three of the twenty studies provided between-group comparisons [5,8,26], twenty studies, including the aforementioned three, were used repeated measures that compared types of HPDs or made comparisons with and without HPDs.

#### 3.2.4. Outcomes

To identify the functions and effects of HPDs, the outcomes of the reviewed studies were classified. Five of twenty studies mainly determined the functions and effects of HPDs by sound attenuation [5,7,20,21,22]. Outcomes of sound localization and speech perception were reported by eight [4,8,9,10,18,23,24,25] and seven [3,11,12,13,19,26,27] of the twenty studies, respectively.

### 3.3. Overall Functions and Effects of Hearing Protection Devices

**Figure 2:**
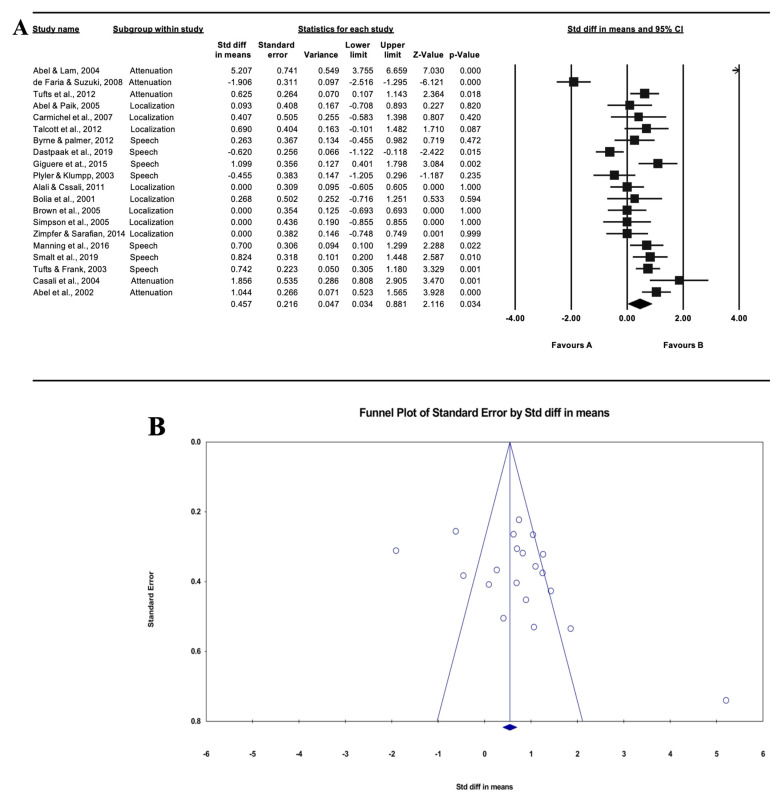
Forest plot of the twenty reviewed studies overall (**A**) and funnel plot of standard error for the standardized difference in means. Asymmetrical graph of funnel plot indicating publication bias (**B**).

The results of effect size for the overall studies with the random effect model are presented in Figure 2A. Overall, the studies showed an SMD of 0.457 (95% CI: 0.034–0.881, *p* < 0.05), indicating that there was a significant functional difference. To identify the publication bias for the reviewed studies, a funnel plot is presented in Figure 2B. Not only was the funnel plot asymmetrical, but also the results of Egger’s regression analysis showed that there was publication bias in the reviewed studies (intercept: 4.159, SE: 2.310, *p* < 0.05). Further, the results of Higgins *I^2^* statistics and Cochran’s Q test showed that heterogeneity was high (*I^2^*: 88.08, Q: 159.369, *p* < 0.001).

### 3.4. Subgroup Analysis

**Figure 3:**
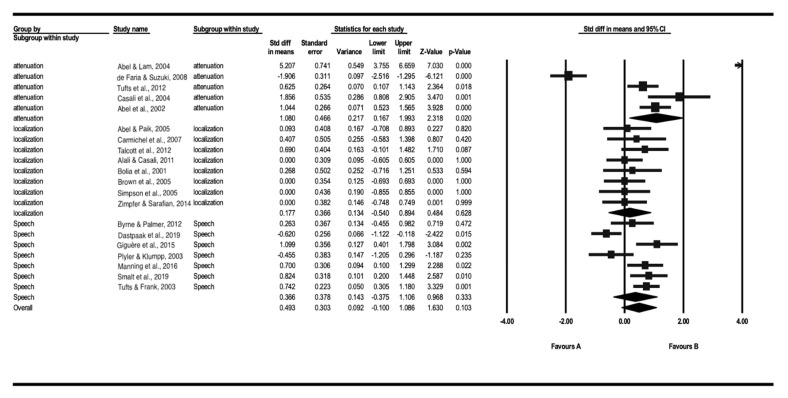
The forest plot of the subgroup analysis for sound attenuation (*n* = 6), sound localization (*n* = 9), and speech perception (*n* = 8).

To identify the results of the meta-analysis more clearly, a subgroup analysis (i.e., attenuation, sound localization, and speech perception) was conducted. For the functions and effects of HPDs, a subgroup analysis was conducted and is presented in Figure 3. First, the effect size of sound attenuation which compared the hearing thresholds for the control (i.e., unoccluded and/or open ear) and an experimental group (i.e., wearing the HPDs) showed 1.080 (95% CI: 0.167–1.993, *p* < 0.05).

Secondly, the subgroup analysis for sound localization included those studies that mainly reported the results for localization ability, such as the percentage correctness, response time, and azimuth error. These results confirmed that the ability of sound localization was not significantly different (SMD: 0.177, 95% CI: −0.540–0.894, *p* = 0.628) in individuals with HPDs compared to individuals without HPDs.

Finally, the studies that reported various speech perception performances (i.e., speech perception in noise, speech intelligibility, and word recognition ability) were classified as a subgroup of speech perception. The results for speech perception demonstrated that HPDs partially support the ability of speech perception, but they do not have any significant effect (SMD: 0.366, 95% CI: −0.375–1.106, *p* = 0.333).

## 4. Discussion

The primary purpose of the current study on systematic review and meta-analysis was to identify the effectiveness of HPDs. Three outcome measures, including sound attenuation, sound localization, and speech perception, were divided into two factors based on function (i.e., sound attenuation) and effect (i.e., sound localization and speech perception). Based on the effect size of a total of twenty reviewed studies, the functions and effects of HPDs that were revealed were significant. Several studies had a value 0 between the lower limit and upper limit of 95% CI and/or a p-value higher than 0.05 which means there were high heterogeneities. These results might have occurred due to the small sample size. In fact, there were no studies that demonstrated a sample size calculation for their study design.

### 4.1. Do HPDs Effectively Attenuate the Level of Intensity of Unwanted Noise?

The major and key functions of HPDs were revealed to be significantly effective, which means the hearing thresholds for the experimental group (wearing the HPDs) increased more than that of the control group (not wearing the HPDs). Abel and Lam reported that both conventional and level-dependent earplugs attenuated the input sounds from 21 to 40 dB and 5 to 22 dB, respectively [20]. This result suggested that individuals who might recommend or willingly wear the HPDs have the choice to select HPDs to meet their purposes and situations. For example, the level-dependent earplugs were regarded as an alternative choice for the hearing-impaired or normal hearing in the case of a relatively not loud situation due to the increased hearing thresholds. According to Casali and colleagues [21], wearing HPDs significantly attenuated the sound more than not using HPDs. They also reported that masked thresholds when wearing HPDs at 85 dBA were more significantly reduced than without having the HPDs condition. These results implied that HPDs could meet the role of their designated purpose.

Again, the HPDs are direct wearing devices, which were originally designated to prevent noise exposure and preserve hearing acuity [32]. Interestingly, Tufts et al. reported the effects of user training and fitting consistency [7]. Their results showed the significant effects of user training. Compared to non-customized earplugs, customized earplugs showed significantly better consistency of attenuation values at frequency ranges of 500, 1000, and 2000 Hz. Moreover, the effects of user training were significant at three testing frequencies (i.e., 250, 500, and 1000 Hz). These results suggest that the function of HPDs, especially for sound attenuation, could be improved and decrease the deviation for trials where individuals tried to wear HPDs. Regardless, to obtain the best benefit from the HPDs, an appropriate fitting method should be required [5] because the uncomfortable feeling of HPDs stems from an inadequate fitting method and/or the type of HPDs and is reported as a problem when wearing HPDs [4].

### 4.2. Does Wearing HPDs Affect Sound Localization Ability for Their Users?

The effects that occurred while wearing the HPDs were analyzed as sound localization ability. Sound localization was defined as a kind of ability, such as percentage correctness, response time, and azimuth error. The results of sound localization when wearing the HPDs could not significantly change the ability of sound localization compared to the condition of no HPDs [4,8,9,10,24]. In other words, wearing the HPDs did not interfere with sound localization, but it did not improve sound localization for their users. The reason why the HPDs failed to improve the ability of sound localization might be due to the distortions of high-frequency spectral cues [4]. Simpson et al. argued that double protection, such as a combination of earplugs with earmuffs, does not help localize the incoming sounds [24]. Simply, in terms of the latest technology of HPDs, active noise reduction HPDs may be better than the HPDs in terms of passive noise reduction. Still, Abel and Paik argued that not only the earmuff is not desirable to use for directionality, but also that the active noise reduction HPDs do not help recover the sound localization ability. In addition, the studies conducted by Carmichel et al. and Talcott et al. compared the two different functions of active and passive noise reduction HPDs and concluded that both noise reduction HPDs unfortunately did not recover the ability to determine sound localization [9,10].

### 4.3. Does Wearing an HPD Affect a User’s Speech Perception Ability?

In view of user ability to have speech perception, similar to sound localization ability, the effect of HPDs was not significant based on the current meta-analysis. Since many of the studies that have reported the function of HPDs for speech perception ability are controversial, we believe that a current decisive and unified analysis is more informative. That is, three of seven studies which were clustered as a subgroup of speech perception argued that to wear HPDs did not improve speech perception ability [3,11,13]. Smalt et al. further reported the effects of HPDs on the listening effort, which was regarded as cognitive resources for auditory tasks [3]. They concluded that the use of HPDs increased the amount of listening effort. This increased listening effort produced cognitive fatigue, especially in noisy circumstances.

On the other hand, three studies posited that wearing HPDs could improve speech perception performance [12,26,27]. Although passive noise reduction HPDs which have a low noise reduction rating (NRR) showed increased speech intelligibility in the presence of background noise [12], active noise reduction HPDs provided substantial benefits for speech recognition performance, especially for the hearing-impaired users [26]. Plyler and Klumpp also suggested that active noise reduction HPDs may be beneficial for individuals with sensorineural hearing loss [19]. Further considering the characteristics and requirements of users who would be recommended and/or mandated for HPDs, Manning et al. reported on the benefits of bone-conduction HPDs for tinnitus patients [27]. They conducted speech recognition tasks under noisy circumstances for tinnitus patients, the hearing-impaired users, and normal hearing listeners, using both air- and bone-conduction HPDs. The authors reported that bone-conduction HPDs exhibited better performances than air-conduction HPDs did in all groups.

## 5. Conclusions

Based on the present systematic review that included meta-analysis, the HPDs performed well for their originally designated function, i.e., sound attenuation, without interfering with finding a location from the sound sources and talk between the workers. It is obvious that HPDs provide great benefits in specific situations and/or for individuals, such as in the presence of high-intensity noise, military soldiers, and occupational workers, b due to the attenuation of HPDs. Furthermore, the performance of sound localization and speech perception were not negatively affected for those users wearing the HPDs.

Although the present systematic review and meta-analysis were conducted with specific purposes, such as the evaluation of major functions and effects (i.e., sound attenuation, sound localization, and speech perception) of HPDs, a comparison between passive and active HPDs was not considered. For example, six articles used passive HPDs, and fourteen articles used active HPDs in the twenty reviewed articles. It is obvious that the mechanisms of passive HPDs and active HPDs are differently enacted, and these types of mechanisms for HPDs should be discussed in further.

In conclusion, because the results of HPDs analysis from many previous studies are controversial, high-level evidence of using HPDs will need to include evidence-based guidelines for those individuals who are recommended and/or mandated to wear HPDs.

## Figures and Tables

**Table 1:** Inclusion criteria for the present study using Participants, Intervention, Control, Outcomes, and Study Designs (PICOS) Strategy.

Parameter	Inclusion Criteria
Participants	Adults 18 years or older with and without hearing loss
Intervention	Functions and effects of hearing protection devices (i.e., comparison of wearing or not wearing hearing protection devices or a comparison of the types of hearing protection devices)
Control	Comparison to control group or repeated measures (experiments with additional purposes)
Outcomes	Outcome measure(s) related to functions and/or effects of hearing protection devices (i.e., sound attenuation, sound localization, and speech perception)
Study Designs	Randomized controlled trials, non-randomized controlled trials, and repeated measures (experiments with additional purposes)

**Table 2:** Analysis of the scientific study validity criteria based on CAMARADES checklists [16].

Study	Scientific Study Validity Criteria						Study Quality Score
	Randomization	Controls	Sample Size Calculation	Publication after Peer Review	Outcome Measure	Statement of Potential Conflict of Interest	
Smalt et al. (2019) [3]	1	0	0	1	1	1	4
Brown et al. (2015) [4]	1	0	0	1	1	1	4
Abel et al. (2002) [5]	1	1	0	1	1	0	4
Tufts et al. (2012) [7]	0	0	0	1	1	0	2
Abel and Paik (2005) [8]	1	0	0	1	1	1	4
Carmichel et al. (2007) [9]	1	0	0	1	1	1	4
Talcott et al. (2012) [10]	1	0	0	1	1	1	4
Byrne and Palmer (2012) [11]	0	0	0	1	1	1	3
Dastpaak et al. (2019) [12]	1	0	0	1	1	1	4
Tufts and Frank (2003) [13]	1	0	0	1	1	0	3
Alali and Casali (2011) [18]	1	0	0	1	1	1	4
Plyler and Klumpp (2003) [19]	1	0	0	1	1	0	3
Abel and Lam (2004) [20]	0	0	0	1	1	0	2
Casali et al. (2004) [21]	1	0	0	1	1	0	3
de Faria and Suzuki (2008) [22]	0	0	0	1	1	0	2
Bolia et al. (2001) [23]	1	0	0	1	1	0	3
Simpson et al. (2005) [24]	1	0	0	1	1	0	3
Zimpfer and Sarafian (2014) [25]	1	0	0	1	1	1	4
Giguère et al. (2015) [26]	1	1	0	1	1	1	5
Manning et al. (2016) [27]	1	1	0	1	1	0	4

1 and 0 refer to “Yes” and “No”, respectively.

**Table 3:** Characteristics and effects of hearing protection devices for all enrolled studies for the participants, intervention, control group, and outcome of each study.

Study	Participants	Test Materials and Conditions	Study Design	Major Function	Outcome Measures	Main Findings	Effects of HPD
Abel et al. (2002) [5]	Twenty-four young adults with normal hearing (aged under 40 years), twenty-four older adults with normal hearing (aged over 40 years), and twenty-four older adults with hearing loss	A total of five ear conditions were adjusted: (1) ears under unoccluded conditions, (2) ears with Class A muffs, (3) ears with muffs on hard hat and air-purifying half-mask respirators, (4) ears with muffs on hard hat and safety glasses, and (5) ears with muffs on hard hat, safety glasses, and respirators. Attenuation measurements were conducted for eight one-third octave noise bands centered from 0.25 to 8 kHz.	Repeated measures	Attenuation	Amount of attenuation (dB SPL)	The results of ANOVA with repeated measures showed significant effects for group [F(5,61) = 4.3, *p* < 0.002], protector condition [F(3,183) = 104.2, *p* < 0.0001], and frequency [F(7,427) = 387.7, *p* < 0.0001]. The interaction of the protector condition by group [F(35,427) = 1.8, *p*< 0.006], protector condition by group [F(21,1281) = 12.2, *p* < 0.0001], protector condition by frequency by group [F(105,1281) = 6.8, *p* < 0.04]. The interaction with protector condition by group was not significant.	Averaged across protector conditions, attenuation showed significant increases at frequency increased from 0.25 to 1 kHz and then remained constant, except for a dip at 6.3 kHz. Averaged across groups and frequencies, the least attenuation was achieved with the muff on hard hat in combination with the glasses and respirator. The muff on hard hat alone condition showed highest attenuation. The range in attenuation in order to the conditions was greater at 0.25 and 0.5 kHz (9 dB) and at its lowest 2 and 3.15 kHz (3–4 dB).
Abel and Lam (2004) [20]	Sixteen adults ages from 21 to 53 years (8 men and 8 women) with normal hearing	The Indoor and Outdoor E-A-R plugs manufactured by the Aero Company were used. The hearing thresholds were measured on nine one-third-octave noise bands (i.e., 0.125, 0,25, 0,5, 1, 2, 3.15, 4, 6.3, and 8 kHz) for three conditions (i.e., unoccluded, indoor plug, and outdoor plug)	Repeated measures	Attenuation	Amount of attenuation (dB).	The results of ANOVA with repeated measures showed significant effects of ear condition, frequency and ear by frequency (*p* < 0.0001). Post hoc comparison showed that the differences in attenuation for the two devices was significant for all testing frequencies (*p* < 0.001 or better).	The attenuation values in the indoor plug increased from 21 dB at 0.125 kHz to 40 dB at 8 kHz. Additionally, the results for the outdoor plug attenuated the thresholds by 5 and 14 dB at 0.5 kHz and 1 kHz.
Abel and Paik (2005) [8]	Twelve young adults (age range—18 to 30 years) and twelve older adults (age range—40 to 55 years) with normal hearing	Three ear conditions (i.e., with and without ANR earmuff operational and unoccluded ear) were used. A total of eight speakers formed eight azimuth angles (i.e., 15, 75, 105, 165, 195, 255, 285, and 345 degrees). For the sound source identification task, three different signals, such as 0.5 kHz, 4 kHz, and broadband noise, were presented.	Repeated measures	Sound localization	Response time (ms) for sound source identification	An ANOVA indicated that there were significant effects for ear condition [F(2,40) = 5.6, *p* < 0.007] and stimulus frequency [F(2,40) = 9.0, *p* < 0.001].	Averaged across the four groups and three stimuli, the mean median response times ranged from 712 ms for ANR On to 805 ms in the unoccluded condition. Averaged across the four groups and three ear conditions, the mean median response times ranged from 667 ms for the broadband noise to 803 ms for the 0.5 kHz stimulus.
Alali and Casali (2011) [18]	Twelve adults (older than 18 years) with normal hearing	Within-subject design (8 HPDs × 2 signals × 2 presentation levels) were adjusted. The HPDs consisted of: (1) unoccluded, (2) foam earplug, (3) pre-molded earplug, (4) flat attenuation HPD, (5) level-dependent HPD, (6) passive earmuff, (7) dichotic sound transmission earmuff, and (8) custom-made diotic sound transmission earmuff. The signals consisted of a standard backup alarm and spectrally modified one. Presentation levels of pink noise were both 60 dBA and 90 dBA.	Repeated measures	Sound localization	Percentage correct (%) of sound identification.	The ANOVA revealed the significant main effects of HPD for a percentage correct localization [F(7,77) = 59.2, *p* < 0.0001], percentage of right–left localization errors [F(7,77) = 42.78, *p* < 0.0001], percentage of front-rear localization errors [F(7,77) = 43.03, *p* < 0.0001], and localization absolute deviation in degrees [F(7,77) = 92.67, *p* < 0.0001]. The interaction between HPD and background noise level on percentage correct localization response was also significant [F(7,77) = 9.51, *p* < 0.0001].	The mean values of percentage correctness localization showed that flat attenuation HPD (83.9%) was the most correct in terms of percentage. Unoccluded (82.2%), pre-molded earplug (81.5%), and passive earmuff followed, but were not significant. The custom-made diotic sound transmission earmuff (66.3%) and foam earplug (64.6%) showed similar percentages of correctness. The dichotic sound transmission earmuff (15.8%) showed the lowest percentage correctness for localization.
Bolia et al. 2001 [23]	Six adults (aged 18 to 34 years) with normal hearing	Three hearing protector conditions (i.e., earplugs, earmuffs, and unoccluded ear) were adjusted factorially with three source elevation conditions (upper hemisphere, peri-horizontal region, and lower hemisphere) for a total of nine experimental conditions.	Repeated measures	Sound localization	Azimuth error (°)	Mean azimuth errors were analyzed using a 3 (hearing protector) × 3 (source elevation) repeated-measures ANOVA, employing the Huynh–Feldt correction, which revealed significant main effects of hearing protector, [F(2,10) = 29.94, *p* < 0.05], and source elevation, [F(2,10) = 19.96, *p* < 0.05], and a Hearing Protector × Source Elevation interaction, [F(4,20) = 10.86, *p* < 0.05].	All simple main effects of the hearing protector factor were statistically significant (*p* < 0.01), implying that at one or more levels of the source elevation factor, performance varies as a function of the HPD factor.
Brown et al. (2015) [4]	Ten normal hearing adults (mean age: 29.5 years)	Four HPDs and one control (unoccluded) were used in the experiment: (1) passive HPD, (2) active HPD, (3) hybrid HPD, and (4) ShotShields HPD.	Repeated measures	Sound localization	Response angle (°)	The ANOVA indicated a significant main effect of device condition [F(4,36) = 13.64, *p* < 0.001] and target angle [F(11,99) = 5.67, *p* < 0.001]. A significant interaction between the device and target angle [F(44,396) = 5.67, *p* < 0.001] was revealed.	All tested HPDs significantly degraded localization performance relative to control (unoccluded) condition. However, post hoc analysis of HPD-related distortions of sound source localization cues demonstrated that the ShotShields produced both the best behavioral performance and the greatest preservation of sound source cues.
Byrne and Palmer (2012) [11]	Fifteen adults with normal hearing (age range—21 to 60 years)	In the four testing conditions, two types of HPDs were used, an electronic hearing protector and conventional passive earmuff. The electronic hearing protector had three different settings (i.e., off, low, and high).	Repeated measures	Speech intelligibility	Correct percentage of HINT test score (%)	The results of repeated-measures ANOVA showed a significant main effect [F(2,28) = 1014.50, *p* < 0.0001] for the SNR condition. The highest scores were obtained under the +5 dB SNR condition, while the lowest scores were obtained under the −5 dB SNR condition. A significant main effect was also found for the earmuff condition [F(3,42) = 57.19, *p* < 0.0001]. Additionally, the interaction effect was significant [F(6,84) = 6.94, *p* < 0.0001].	For the types of HPDs, passive muff showed the highest correctness percentage for the all SNR conditions. While the off and low settings of electronic hearing protector showed similar correctness percentages, and the high setting of hearing protector showed the lowest correctness percentage, regardless of the SNRs.
Carmichel et al. (2007) [9]	Eight normal hearing listeners (aged 20 to 40 years)	Four stimuli (i.e., two successive transient clicks of a semiautomatic handgun being loaded, the double ring of a telephone with a mechanical ringer, and electronically generated FM tone bursts at 0.5 and 4 kHz) and four HPDs (i.e., electronic earmuffs, dynamic level compression protectors, action ear sport, and unoccluded) were used.	Repeated measures	Sound localization	Localization performance (%) and response time (sec)	Differences in localization among the conditions were significant for the firearm [F(3,21) = 15.30, *p* < 0.05], telephone [F(3,21) = 15.40, *p* < 0.05], 4 kHz tone burst [F(3,21) = 1.13, *p* < 0.05] stimuli, but not for the 0.5 kHz tone burst [F(3,21) = 1.13, *p* = 0.36]. The mean response time showed that there were differences in the conditions for the broadband stimuli (firearm, [F(3,21) = 6.15, *p* < 0.05], telephone [F(3,21) = 5.21, *p* < 0.05].	The mean correct responses for localization to broadband stimuli in the unoccluded condition were 97.2% (firearm) and 98.6% (telephone). Mean scores for localization to these stimuli ranged from 67.4 to 54.2% correctness in the HPD conditions when averaged across all locations. When averaged across all stimuli and locations, the mean response time for correct responses in the unoccluded condition (mean: 1.58s, SD: 0.76) was not different from the mean response time to errors (mean: 1.64s, SD: 0.81). In the HPD conditions, the mean response time to incorrect response was less (mean: 1.88 s, SD: 0.89) than to correct responses (mean: 1.90 s, SD: 0.81) but these differences were not significant.
Casali et al. (2004) [21]	Ten adults with normal hearing, wuth age ranging from 18 to 49 years	The signal stimulus was a standard vehicle back-up alarm at a presentation level of 85 and 100 dBA. The experimental conditions consisted of four different ear conditions (i.e., earplug, passive earmuff, ANR earmuff, and unoccluded ear).	Repeated measures	Attenuation	Mean masked thresholds (dBA)	In the comparisons of HPD analyzed by ANOVA with repeated measures, interactions including ear condition x noise level [F(2,18) = 19.38, *p* = 0.0001], ear condition x noise spectrum [F(2,18) = 8.51, *p* = 0.0053] were significant. In comparisons of HPDs and an unoccluded condition, there were no statistically significant interactions for ear condition x noise spectrum. However, the main effects of the ear condition at 85 dBA [F(3,27) = 7.91, *p* = 0.0006] were significant.	Given 100 dBA noise, earplugs showed significantly lower masked thresholds (91.9 dBA) than either the ANR muff (93.8 dBA) or the passive earmuff (95.5 dBA). Additionally, the ANR muff produced significantly lower masked thresholds than the passive muff did.
Dastpaak et al. (2019) [12]	Thirty-two adults (mean age: 26.12 years) with normal hearing	Three ear conditions (unoccluded, HPD with 25 NRR, and HPD with 32 NRR) and three SNR condition (−10, 0, and +10 dB) were used.	Repeated measures	Speech intelligibility	Averaged intelligibility score (%)	The results showed maximum speech intelligibility without HPDs and without noise and minimum average of speech intelligibility when the background noise was greater than the −10 dB SNR.	For the comparisons of types of HPDs, 32 of NRR HPD showed lower averaged intelligibility scores in all testing SNR conditions. While the without HPD condition showed 8.13% (SD: 9.11), 35.44% (SD: 19.11), and 74.94% (SD: 7.67) for three SNR conditions, HPD with 32 NRR showed 11.38% (SD: 9.64), 43.25% (SD: 17.54), and 78.06% (SD: 9.57). The HPD with 25 NRR showed highest average intelligibility score for −10 dB SNR (mean: 22.75%, SD: 12.04), 0 dB SNR (mean: 55.31%, SD: 14.41), and −10 dB SNR (mean: 78.38%, SD: 13.39).
de Faria and Suzuki (2008) [22]	Thirty adults with normal hearing (age range—20 to 58 years)	Two HPDs with 21 and 17 dB NRR, respectively, were used to measure attenuation.	Repeated measures	Attenuation	Noise attenuation (decibels)	The mean noise attenuation for 60 ears at all frequencies was lower than the manufacturer’s rating. No ear achieved the expected attenuation, except at 500 Hz (35 dBNA).	The observed noise attenuation measurements at each frequency were: (1) 500 Hz (mean: 22.8 dB, SD: 3.8), (2) 1000 Hz (mean: 23.4 dB, SD: 3.7), (3) 2000 Hz (mean: 27.3 dB, SD: 4.5), and (4) 4000 Hz (mean: 29.4 dB, SD: 4.3).
Giguère et al. (2015) [26]	A total of forty-five adults (age of 23 to 81 years) participated. There were four groups based on t hearing loss: (1) normal hearing (n = 12), (2) slight-to-mild (n = 12), (3) mild-to-moderate (n = 12), and (4) moderate/severe (n = 9)	Three ear conditions were used (unoccluded and two HPDs). In the HPDs, one was an analogue device, and the other was a digital device. Two noises (95.3 dBA and 89.5 dBA) were used.	Repeated measures	Speech intelligibility	Speech recognition (% word recognition)	In a comparison of protected versus unprotected speech recognition scores by hearing loss, use of the higher level-dependent function almost fully restored speech recognition to unprotected values or provided significant improvements over an unprotected performance for all participants.	The passive HPD showed a mean decrease in performance of 27–29% across the noises compared to the unoccluded condition. However, when the level-dependent function was activated, it yielded average overall benefits of 11–15% and 23–24% across the two noises, respectively. The active HPD showed a mean decrement in performance of 17–25% compared to unprotected listening across the two noises. Unlikely for the passive HPD, an active HPD with activation of level-dependent function showed only a small effect on performance and a mean benefit of 6–7%.
Manning et al. (2016) [27]	A total of 47 adults (19 with normal hearing, 15 with SNHL, and 13 with SNHL and tinnitus).	A total of 300 items consisting of six lists were used. The background noise was presented in three different SNR conditions (i.e., quiet, −6 and −12 dB). Two types of TCAPS (i.e., in-ear headset for air-conduction, and headset for bone-conduction) and two types of talker (male and female) were used.	Repeated measures	Speech intelligibility	Mean rationalized arcsine units (corrected)	The mean rationalized arcsine units measured for each of the TCAPS under test were marginally, but significantly better for the bone-conduction headset [F(1,528) = 29.90, *p* < 0.01]. For the types of hearing loss, the effects of hearing loss were found to be statistically significant [F(2,528) = 3.74, *p* < 0.024]. There was no significant interaction of hearing loss with TCAPS [F(2,528) = 0.99, *p* = 0.372]. In the SNR conditions, performance increased, as the SNR increased [F(2,528) = 1881.47, *p* < 0.01].	The normal hearing listeners performed best, and SNHL listeners performed worst. The degrading effect of noise on speech recognition was approximately the same for the normal hearing and hearing impaired listeners; there was no measurable benefit for either TCAPS in noise.
Plyler and Klump (2003) [19]	Fourteen females (age 21 to 24 years) with normal hearing	A total of three ear conditions including unoccluded were used. In the HPDs, no HPDs which was custom acoustic HPD and custom electronic HPD were used. For the speech perception test, two levels of noise (75 and 90 dB SPL) were used. The HINT sentence lists were used as the stimuli.	Repeated measures	Speech intelligibility	Relative HINT score	The analysis revealed a significant main effect for HPD [F(1,13) = 10, *p* < 0.05]. However, main effects for sentence presentation level [F(1,13) = 1, *p* > 0.05] were significant. The interaction between sentence presentation level x HPD [F(1,13) = 2, *p* > 0.05] was not significant. These results indicated that communication during noise was significantly better when utilizing the acoustic HPD than when utilizing the electronic HPD at each sentence presentation level.	The averaged relative HINT scores indicated that the acoustic HPD showed 1.1 (SD: 1.9) relative HINT score which was higher than the electronic HPD (mean: −0.4, SD: 1.2) at 75 dB SPL. In the 90 dB SPL condition, acoustic HPD (mean: 0.1, SD: 1.1) also showed higher relative HINT score than that for the electronic HPD (mean: −0.6, SD: 1.3).
Simpson et al. (2005) [24]	Seven adults (age 18 to 39 years) with normal hearing	Four ear conditions (i.e., no HPD (unoccluded), foam earplugs, earmuffs, combination of earplugs and earmuffs) and five auditory cue conditions (four HPDs condition and no cue condition) were used. Stimuli were visual targets with continuous broadband (70 Hz to 16 kHz) pink noise.	Repeated measures	Sound localization	Response accuracy (%) and search time (sec)	For response accuracy, repeated-measures ANOVA revealed that neither of the main effects nor the interaction was found to be statistically significant (*p* > 0.05). However, for search time, repeated ANOVA showed significant main effects on the auditory condition [F(4,24) = 239.11, *p* < 0.05] and set size [F(2,12) = 328.75, *p* < 0.05]. The interaction between auditory condition and set size [F(8,48) = 93.84, *p* < 0.05] was significant.	The unoccluded condition had the lowest search time, regardless of the set size. Earmuff and earplug conditions showed similar search times as a function of set size. The combination of earplugs and earmuffs had a higher search time than for other ear conditions.
Smalt et al. (2019) [3]	Thirteen adults average age of 31 years with normal hearing, except for one subject which showed 30 dB HL in one ear	A total of four HPDs, two passive types and two active types, were used. For the speech intelligibility test, Modified Rhyme Test using three background noises (i.e., 60, 75, and 80 dBA) was used.	Repeated measures	Speech intelligibility	Percentage correct (%)	A two-way ANOVA demonstrated that there were significant main effects for HPD [F(4,48) = 3.716, *p* = 0.010] and noise level [F(2,24) = 1737, *p* < 2 × 10^−16^. However, the interaction between HPD and noise level was not significant. For the results of the post hoc test, only the HPD pair showed a significant difference between active HPDs (*p* = 0.017).	Speech intelligibility was somewhat reduced for all the hearing protectors, but the differences induced by increasing the background noise level were much greater than the differences among the HPDs for a single noise level.
Talcott et al. (2012) [10]	Thirteen adults age 22 to 54 years with normal hearing	Five ear conditions (i.e., unoccluded, earplug with 21 dB NRR and gain switch, ear tips with 21 dB NRR, earplugs with 7 dB NRR, earmuff with 21 dB NRR) and two noise conditions (i.e., 45–50 dBA ambient noise and 82 dBA diesel truck noise) were used.	Repeated measures	Sound localization	Percentage correctness response (%) and mean response time (sec)	The ANOVA showed a significant main effect of the listening condition (F = 17.22, *p* < 0.0001). For the mean response time, the results of ANOVA showed that there was a significant main effect of the listening condition (F = 11.11, *p* < 0.0001).	The percent of correct responses was significantly lower for the earmuff (21 ± 10%) than all listening conditions and significantly greater for the unoccluded ear (55 ± 17%) than all listening conditions. No other differences were significant between the HPD conditions. In addition, the mean response time was significantly higher for the earmuff condition (2.9 ± 1.4 s) than for all other HPDs and the unoccluded condition.
Tufts et al. (2012) [7]	Thirty adults with normal hearing (mean age: 21 years, SD: 2.7)	Two types of HPDs were used (i.e., custom-molded and non-custom earplugs). For five measurements, the results of trained and untrained groups were compared.	Repeated measures	Attenuation	Mean per-frequency attenuation (dB)	Repeated-measures of ANOVA conducted on mean PAR showed the training to be statistically significant [F(1,29) = 11.77, *p* = 0.002], but neither earplug type [F(1,29) = 1,91, *p* = 0.178] nor the interaction of earplug type x training [F(1,29) = 0.29, *p* = 0.596] was statistically significant.	Mean per-frequency attenuation and mean PAR for each earplug type and training condition showed that the mean PAR of untrained non-custom plug produced 3.2 dB less attenuation than the trained non-custom plug condition. For the custom plug, the untrained group showed 4.1 dB less mean PAR than the trained group. For the frequency comparison, the trained group showed higher attenuation values than the untrained group did for both custom and non-custom plugs.
Tufts and Frank (2003) [13]	Thirty-two adults (mean: 25.4 years, SD: 4.9) with normal hearing	Two types of HPDs (i.e., custom-molded foam ear plug and pre-molded earplug) were used. As stimuli, the 12 passages from the 20 passages of the Speech Intelligibility Rating test were chosen.	Repeated measures	Speech intelligibility	Signal-to-noise ratio (dB)	In the SNR analysis, the main effects of background noise [F(4,112) = 3041.646, *p* < 0.001] and ear condition [F(1,28) = 4965.188, *p* < 0.001] were significant. Further, the interactions of background noise x ear condition [F(4,112) = 60.568, *p* < 0.001] and background noise x ear condition x sex [F(4,112) = 2.776, *p* = 0.034] were significant.	For background noise at 100 dB SPL, the overall speech levels were 84.4 dB for an unoccluded condition, and 71.9 dB and 74.3 dB for the custom-molded and pre-molded ear plugs. The corresponding SNRs were −15.6, −28.1, and −25.7 dB.
Zimpfer and Sarafian (2014) [25]	Twenty listeners (age 24–51 years) with normal hearing	Five HPDs (four earplugs and one earmuff) were used where two were passive HPD and three were active HPD.	Repeated measures	Sound localization	Number of correct responses and number of confusions	Two-way repeated-measure ANOVA revealed the significant main effect of the test condition factor [F(2.9,10.6) = 68.33, *p* < 0.001].	The unoccluded condition showed a significantly higher number of correct responses than any other HPDs conditions. Moreover, the active systems yielded lower scores (53 and 40%) than did the passive systems (63%).

## Data Availability

The datasets used and/or analyzed for current study are available from the corresponding author on reasonable request.
